# Validity and reliability of the semi-quantitative self-report Home Food Availability Inventory Checklist (HFAI-C) in White and South Asian populations

**DOI:** 10.1186/s12966-016-0381-y

**Published:** 2016-05-04

**Authors:** Maria Bryant, Madison LeCroy, Pinki Sahota, Jianwen Cai, June Stevens

**Affiliations:** Clinical Trials Research Unit, Leeds Institute of Clinical Trials Research, University of Leeds, Leeds, LS2 9JT UK; Bradford Institute of Health Research, Bradford Royal Infirmary, Bradford, BD9 6RJ UK; Department of Nutrition, Gillings School of Global Public Health, University of North Carolina, Chapel Hill, 27599 USA; School of Health and Well-being, Leeds Beckett University, Leeds, LS1 3HE UK; Department of Biostatistics, Gillings School of Global Public Health, University of North Carolina, Chapel Hill, 27599 USA; Department of Epidemiology, Gillings School of Global Public Health, University of North Carolina, Chapel Hill, 27599 USA

**Keywords:** Home, Food, Availability, Measure, Validation, Reliability, South Asian, Inventory

## Abstract

**Background:**

Despite interest in the importance of the home food environment and its potential influence on children’s diets and social norms, there remain few self-report checklist methods that have been validated against the gold standard of researcher-conducted inventories. This study aimed to assess the criterion validity and reliability of the ‘Home Food Availability Inventory Checklist’ (HFAI-C), a 39-item checklist including categories of fruit, vegetables, snacks and drinks.

**Methods:**

The HFAI-C was completed by 97 participants of White and Pakistani origin in the UK. Validity was determined by comparing participant-reported HFAI-C responses to data from researcher observations of home food availability using PABAK and weighted kappa statistics. The validity of measuring the amount of items (in addition to presence/absence) available was also determined. Test-retest reliability compared repeated administrations of the HFAI-C using intra-class correlation coefficients.

**Results:**

Validity and reliability was fair to moderate overall. For validity, the average category-level PABAK ranged from 0.31 (95 % CI: 0.25, 0.37) for vegetables to 0.44 (95 % CI: 0.40, 0.49) for fruits. Assessment of the presence/absence of items demonstrated higher validity compared to quantity measurements. Reliability was increased when the HFAI-C was repeated close to the time of the first administration. For example, ICCs for reliability of the measurement of fruits were 0.52 (95 %CI: 0.47, 0.56) if re-administered within 5 months, 0.58 (95 % CI: 0.51, 0.64) within 30 days and 0.97 (95 %CI: 0.94, 1.00) if re-administered on the same day.

**Conclusions:**

Overall, the HFAI-C demonstrated fair to moderate validity and reliability in a population of White and South Asian participants. This evaluation is consistent with previous work on other checklists in less diverse, more affluent populations. Our research supports the use of the HFAI-C as a useful, albeit imperfect, representation of researcher-conducted inventories. The feasibility of collecting information using the HFAI-C in large, multi-ethnic samples can facilitate examination of home food availability in relation to exposures such as ethnicity and outcomes including behavioural, social and health outcomes. Future work using the HFAI-C could provide important insights into a modifiable influence with potential to impact health.

**Electronic supplementary material:**

The online version of this article (doi:10.1186/s12966-016-0381-y) contains supplementary material, which is available to authorized users.

## Background

Measuring the home food environment and understanding the potential differences in the availability of foods and drinks in homes of different cultures and populations permits a greater knowledge of the causes of energy over-consumption and may support the development of effective obesity prevention and treatment interventions. The majority of work in the area of home food availability has used self-report checklists in which parents/caregivers are asked to report whether foods are present or absent. Despite the increased interest in the importance of the home food environment in recent years [1–5], few researchers have validated self-report checklist methods against the gold standard of researcher-conducted exhaustive home food availability inventories and none within South Asian populations [3, 6–10]. Exhaustive researcher-conducted inventories of all foods in the home provide data that describe the total amounts of foods and nutrients available. However, this approach is rarely used due to the burden of collecting and analysing exhaustive data from within family homes [5].

As food selection is highly linked to culture and ethnicity, it is likely that the foods in the home differ in ethnically and socially distinct households. It is also possible that reporting of foods in the home differs by culture and ethnicity. As such, consideration is also required of the population characteristics when conducting a validation of home food availability checklists. Work investigating home food availability in minority populations has thus far included validity examination in African American, Somali and Hispanic populations [11, 12]. There remains a lack of evaluation of the validity and reliability of home food availability checklists in other populations, including those of South Asian origin.

The current study aimed to assess the validity and reliability of a brief 39-item home food availability checklist; the ‘Home Food Availability Inventory Checklist (HFAI-C)’ in a sample of White British and South Asian populations in the UK. As the precision to which a checklist can assess the amount of foods in the home (in contrast to assessment of their presence only) is not known, we also aimed to determine the degree to which a self-report checklist can accurately estimate the quantity of foods available in homes by comparing validity and reliability from dichotomous outcome data (i.e. food/drink items present or absent) to data from varying levels of quantity (i.e. creating quartiles and tertiles of the quantity of foods available).

## Methods

### Sample

Participants completing the checklist were recruited from ‘Born in Bradford 1000’ (BiB1000) [13]; a nested cohort study within the ‘Born in Bradford’ (BiB) study [14]. In brief, BiB is a longitudinal multi-ethnic birth cohort of 12,453 women who were recruited during pregnancy if they were registered to give birth in the Bradford Royal Infirmary (resulting in 13,776 babies). Participants were almost exclusively of White or South Asian ethnicity. This cohort aims to examine environmental, psychological and genetic factors that impact health and development perinatally and during childhood [14]. The BiB1000 nested population was not a random sample. Instead, all mothers recruited to the full BiB study between August 2008 and March 2009 who had completed the baseline questionnaire were approached to take part in BiB1000 during their routine 26–28 week glucose tolerance test (GTT) [13]. This nested cohort of 1736 mothers aimed to understand the predictors and influences of pregnancy and early life health-related behaviours specifically to inform the development of a culturally specific childhood obesity prevention intervention. BiB1000 characteristics are similar to that of the full BiB cohort [14]; with a similar distribution of age, ethnicity, and marital status (See Table 1). All BiB1000 participants were asked to complete the HFAI-C and a sub-sample of 100 mothers were opportunistically recruited to have researcher-conducted home food availability inventories conducted around the time of their 18 month BiB1000 assessment (i.e. all participants completing their 18 month assessment were invited to take part, with the first 100 consenting included in the study). Inventories were then scheduled to occur as soon as possible following the 18 month assessment.

**Table 1:** Demographic characteristics for analytic sample (*n* = 97)

	HFAI (*n* = 97)		BiB (*n* = 13199)		BiB1000 (*n* = 1707)	
	n	%	n	%	n	%
Maternal age						
< 25	20	21.1	4678	35.4	612	35.9
25–29	36	37.9	4275	32.4	562	32.9
30–34	17	17.9	2749	20.8	325	19.0
≥ 35	22	23.2	1497	11.3	208	12.2
Missing	2	2.0	-	-	-	-
Maternal ethnicity						
White British	46	47.4	4307	32.63	652	38.2
Pakistani	41	42.3	4938	44.9	808	47.3
Other	10	10.3	1643	12.5	247	14.5
Missing	-	-	2311	17.5	-	-
Maternal education ^a^						
None	13	13.7	2356	17.9	375	22.0
School	33	34.7	3361	25.5	556	32.6
Further	10	10.5	1563	11.8	233	13.7
Higher	30	31.6	2772	21.0	404	23.7
Other/unknown	9	9.5	828	6.3	133	7.8
Missing	2	2.0	2319	17.6	6	0.4
Maternal marital status						
Married	72	75.8	7442	56.4	1194	69.9
Divorced	1	1.1	230	1.7	27	1.6
Single	22	23.2	3227	24.5	484	28.4
Missing	2	2.0	2300	17.4	2	0.1
Number of persons in household						
1	2	2.1	220	1.7	27	1.6
2	21	22.1	2506	19.0	384	22.5
3	25	26.3	2659	20.2	413	24.2
4	20	21.1	1928	14.6	287	16.8
≥ 5	27	28.4	3584	27.2	594	34.8
Missing	2	2.1	2302	17.4	2	0.1
Maternal BMI^a^ (Mean, SD)	26.4	5.9	26.0	5.7	25.9	5.7

^a^ School education (up to age 16 years); Further (e.g. A’levels, Senior high school, Diploma, general education certificate); Higher (university certificate, including bachelor and higher)

There were no language restrictions for study eligibility and multi-lingual staff were trained to collect data in homes in which the parents were unable to speak English. All questionnaires were transliterated into Urdu and Mirpuri language, as the majority of Pakistani populations residing in Bradford are of Mirpuri origin and one of the official languages of Pakistan is Urdu.

This study was conducted according to the guidelines of the Declaration of Helsinki and all procedures involving human subjects/patients were approved by the Bradford Research Ethics Committee (07/H1302/112). Written or verbal (for mothers unable to read and/or speak English) informed consent was obtained from all participants. Verbal consent was witnessed and formally recorded.

### Measures

#### Home food availability inventory checklist

The Home Food Availability Inventory Checklist (HFAI-C) was modelled after the Healthy Home Survey [10] and informed by data from a study of home food availability that used an exhaustive barcode scanning method [15]. These studies provided information on which foods were commonly available in the home in addition to providing a range of available sizes for each item. The checklist was intended for use in large cohort studies and therefore was designed so that it did not incur a high level of burden on participants. Foods and drinks were restricted to a list of 39-items that were available within the categories of 1. Fruits (16-items including fresh, dried and canned); 2. Vegetables (12-items including fresh and canned; 3. Snacks (7-items including savoury (e.g. salted nuts) and sweet (e.g. cake)); and 4. Drinks (4-items including regular and diet fizzy drinks, sports drinks and fruit drinks). These were chosen for study because; 1. they are often the target of obesity interventions [16], 2. there is some evidence that their intake is related to obesity in children [17–19] and/or, 3. early literature indicates a relationship between availability in the home and either diet [20, 21] or obesity [22].

The HFAI-C instructs participants to report the maximum availability of each food and drink item in their homes over the previous 7 days. Rather than using a dichotomous response option of ‘present’ or ‘absent’, four categories of response options were generated to enable an estimation of the quantity of foods within the home (absent, small amount, medium amount, large amount). These response options were not intended to precisely quantify the exact amount of foods, but to rank availability. A range of the quantities within each response option was provided based on the distribution of sizes of foods/packages that were previously available from our Universal Product Code (UPC) scanning study [15] and on the usual packaging for purchase.

The HFAI-C was administered as part of a larger questionnaire, which was completed by all BiB1000 participants during cohort assessments when infants were 18 and 36 months of age. For the purposes of the test re-test reliability analyses, checklist data from the 18-month assessment have been used (planned as close to the the time of the researcher-conducted inventorie as possible, but with any permitted). A full questionnaire pack was completed for each infant, so that mothers with multiple births were asked to complete the questionnaire pack multiple times (e.g. two times for twins). Data from three parents were incomplete and as such, the validation analysis sample consisted of 97 parents. Two of these 97 parents included twins but only one child was randomly allocated for comparisons between researcher completed and parent self-report checklists. Three samples were used for the reliability testing including: (1) Full sample, independent of timing between repeated administrations, excluding twins (*n* = 95); (2) Reduced sample with repeated measures conducted within 30 days, excluding twins (*n* = 30); and (3) Twin data taken from an additional BiB1000 sample in which parents were asked to complete separate questionnaires for both infants on the same visit (*n* = 15). This latter sample was included to explore reliability estimates that were independent of shopping and consumption behaviours. Since the HFAI-C was administered as part of a larger questionnaire pack, the timing between repeated administrations of the HFAI-C for parents with twins was approximately 1 h. This reduces the likelihood of parents simply copying responses from one administration to the other. Further, questionnaires were researcher administered and data were entered directly on to an electronic table by the researcher.

### Researcher-conducted food availability inventories

Inventories were conducted when infants were approximately 18 months old using a standardised protocol based on well-established methodologies from previous research [10, 15, 23] in which researchers measured *all* foods from *all* food storage areas in participants’ homes within the higher categories of fruits (with sub-categories: fresh, tinned, dried and frozen), vegetables (with sub-categories fresh, tinned and frozen), snacks (with sub-categories: crisps/tortillas, biscuits, salted nuts, chocolate, sweets, cakes and ice-cream) and drinks (with sub-categories sugar-sweetened and sugar-free). Within each sub-category, open ‘exhaustive’ data were collected rather than using a pre-defined checklist of items. This method requires researchers to remove all items from one storage location at a time and only replace them after they have been recorded; ensuring that all relevant items are included. Exhaustive data from 836 food and drink items that were identified were grouped into 215 individual food and drink types. For example, a ‘packet of chocolate digestive biscuits’ was grouped as ‘biscuits with chocolate topping’ within the sub-category of ‘Biscuits/Sweet snacks’ (under the higher category of snacks). Similarly, all crisps that were made with corn were assigned to the group of ‘tortillas’ within ‘Crisps/Savoury snacks’ and ‘red grapes’ and ‘green grapes’ were grouped as ‘grapes’ within the sub-category of fresh fruits (under the higher category of fruits). For the purpose of these analyses, 1 handful of fresh produce represented 1 serving/cup. Other fresh produce that were recorded as whole units (e.g. melons) were converted to the number of adult size servings by a nutritionist (MB) using standards provided by Self Nutrition Data (http://nutritiondata.self.com/facts) and USDA National Nutrient Database for Standard Reference Release 27 (http://ndb.nal.usda.gov/ndb/foods). A repeated administration of the HFAI-C was also completed by participants in the nested sample of 100 homes on the same day as the researcher-conducted inventories.

### Other measures

Demographic data were obtained from the full BiB cohort during recruitment by self-report (26–28 weeks of pregnancy) [13, 14]. Measures relevant to these analyses include: household structure, marital status, educational status (as a proxy for socio-economic status), and ethnicity.

### Analysis

Criterion validity of the HFAI-C was assessed using the researcher-conducted inventory as the gold standard, with the individual checklist questions matched to food/drink item(s) from the researcher-conducted inventories. Agreement was assessed using kappa statistics, sensitivity (proportion of homes with a given food/drink identified as present by the researcher that were correctly identified as present by the participant) [24] and specificity (proportion of homes with a given food/drink identified as absent by the researcher that were correctly identified as absent by the participant) [8]. Kappa statistics were calculated for responses grouped into two (items present/absent), three (items absent or available in small and medium/large amounts), and four (items absent or available in small, medium and large amounts) categories. The medium and large response categories were combined for the tertile categorization given the small number of responses in the large category.

To adjust for differences in response prevalence and for bias between the researcher and participant, prevalence- and bias- adjusted kappa (PABAK) [24] was calculated in addition to Cohen’s kappa [25] for the dichotomized responses. Cicchetti-Allison linear-weighted kappa [26] was used to examine concordance between the reported and observed responses with the data divided into tertiles and quartiles Weights (*w*) were thus assigned according to the participant category (*i*) and the researcher category (*j*), such that: *w*_*ij*_ = 1 – [|*C*_*i*_ – *Cj*|/(*C*_*C*_ – *C*_1_)], where *C*_*i*_ is the score for *i*, *C*_*j*_ is the score for *j,* Cc is the score for category C (the number of categories), and *C*_*1*_ is the score for the first category. For these analyses, the number of categories equalled either four (quartiles) or three (tertiles). Scores were assigned to each closed-ended category using the median of the range in each respective category. The response category indicating the largest amount was open-ended. For this category, we assigned a score that was 1.5 times the total range of the other responses. As opposed to PABAK, the weighted kappa approach allows for differential weighting according to the level of disagreement between reported and observed responses.

Single-measures intra-class correlation coefficients (ICC) [27] were used to examine test-retest reliability across parent completion of the HFAI-C within 5 months, 30 days and repeated on the same day. Two-way random effects models with measures of absolute agreement [28] were used to assess the dichotomized responses using the INTRACC macro created by Hamer for SAS [29]. For a more detailed account of psychometric methods, see Nunnally [30].

Category-level kappa statistics and ICCs were assessed by collapsing all checklist and researcher responses for all items in a given food/drink category into two variables: an HFAI-C response and a researcher response. Corresponding confidence intervals were obtained using bootstrap resampling method by sampling with replacement from the sub-cohort of 100 mothers 1000 times and then calculating the appropriate statistics on each resulting dataset. The 2.5 and 97.5 percentiles of the distributions of kappa statistics were reported as the bounds of the confidence intervals.

For Cohen’s kappa and weighted kappa interpretation purposes, we used the guidelines proposed by Landis and Koch: < 0.00 = poor, 0.00 – 0.20 = slight, 0.21 – 0.40 = fair, 0.41 – 0.60 = moderate, 0.61 – 0.80 = substantial, and 0.81 – 1.0 = almost perfect [31]. PABAK statistics have been similarly interpreted in the context of existing literature (akin to kappa statistics), although it is recognised that descriptive terms like ‘moderate’ and ‘fair’ are not always universally accepted. Because Cohen’s kappa is mathematically equivalent to the weighted kappa in the case of only two categories, Cohen’s kappa as opposed to PABAK was used for comparison purposes. ICCs were interpreted according to guidelines proposed by Shrout: <0.10 = virtually none, 0.11 – 0.40 = slight, 0.41 – 0.60 = fair, 0.61 – 0.80 = moderate, and 0.81 – 1.0 = substantial [32]. All above analyses were repeated for the White British and Pakistini origin subgroups to explore potential differences by ethnicity. All statistical analyses were performed using SAS version 9.4 (SAS Institute, Inc., Cary, North Carolina).

## Results

### Sample characteristics

Table 1 presents demographic data collected at baseline from the 97 participants, who had complete HFAI-C data. The analytic sample was predominately of White British (47 %) or Pakistani origin (42 %). A high proportion of mothers were between the ages of 25 and 29 years (38 %) and were married (76 %). The level of educational attainment was diverse, with 14 % having not completed any education and 32 % having completed some university education. At least 2 persons (of all ages) lived in nearly all households (98 %). These characteristics are similar to those of the full BiB cohort (e.g. which has 45 % participants of Pakistani origin) [14].

### Criterion validity

The criterion validation results are shown for individual food/drink items and by the four food/drink categories (fruits, vegetables, snacks, and drinks) for the dichotomized responses (presence/absence) (Table 2). Items within each category were ordered in descending order according to participant-reported prevalence. Among fruits and vegetables, participants most commonly reported apples, bananas, and carrots as being present over the previous week. For snacks and drinks, participants most commonly reported that biscuits and fizzy drinks had been available respectively. Across all food/drink items, sensitivity (range: 0.49–1.00) tended to be higher than specificity (range: 0.14–0.88).

**Table 2:** Criterion validity comparing researcher administered and participant reported data for analytic sample using dichotomized responses (presence/absence) (*n* = 97)

		Prevalence (%)				
Item	*n*	Participant	Researcher	Sens	Spec	PABAK (95 % CI)
Apples	97	95	71	1.00	0.18	0.53 (0.44, 0.61)
Bananas	96	95	63	1.00	0.14	0.35 (0.26, 0.45)
Grapes	94	84	43	1.00	0.28	0.17 (0.07, 0.27)
Oranges	95	75	34	1.00	0.38	0.18 (0.08, 0.28)
Dried fruit	95	64	57	0.80	0.56	0.39 (0.30, 0.48)
Berries	93	60	18	1.00	0.49	0.16 (0.06, 0.26)
Pears	95	58	31	0.97	0.59	0.41 (0.32, 0.50)
Canned fruit, water/juice	95	41	21	0.65	0.65	0.31 (0.21, 0.40)
Melon	91	41	18	1.00	0.72	0.54 (0.45, 0.63)
Canned fruit, syrup	95	37	31	0.66	0.76	0.45 (0.36, 0.54)
Plums	95	33	9	1.00	0.74	0.54 (0.45, 0.62)
Kiwis	95	32	14	0.85	0.77	0.56 (0.47, 0.64)
Peaches	93	27	9	0.75	0.78	0.55 (0.46, 0.63)
Pineapple	96	25	4	1.00	0.78	0.58 (0.50, 0.66)
Fruit salad	96	22	0	0.00	0.78	0.56 (0.48, 0.65)
Grapefruit	96	15	3	1.00	0.88	0.77 (0.71, 0.83)
TOTAL FRUIT		50	26	0.85	0.59	0.44 (0.40, 0.49)
Carrots	96	89	50	0.98	0.21	0.19 (0.09, 0.29)
Peas	94	87	51	0.98	0.24	0.23 (0.14, 0.33)
Tomatoes, fresh	97	84	66	1.00	0.48	0.65 (0.57, 0.73)
Lettuce	95	75	40	1.00	0.42	0.31 (0.21, 0.40)
Greens	94	71	19	0.94	0.34	-0.09 (-0.19, 0.02)
Tomatoes, can	93	71	56	0.94	0.59	0.57 (0.49, 0.65)
Sweet corn	95	68	33	0.97	0.45	0.24 (0.14, 0.34)
Broccoli	93	58	25	0.87	0.51	0.20 (0.10, 0.30)
Other vegetables, fresh	83	48	95	0.49	0.75	0.01 (-0.10, 0.12)
Cabbage	95	42	15	1.00	0.68	0.45 (0.36, 0.54)
Green beans	97	40	12	0.67	0.64	0.28 (0.18, 0.37)
Celery	93	29	12	1.00	0.80	0.66 (0.58, 0.73)
TOTAL VEGETABLES		64	39	0.90	0.51	0.31 (0.25, 0.37)
Biscuits (cookies)	96	94	84	0.98	0.27	0.73 (0.66, 0.80)
Crisps (chips)	96	92	82	0.95	0.24	0.65 (0.57, 0.72)
Chocolate	94	83	47	0.93	0.26	0.15 (0.05, 0.25)
Sweets (candies)	95	65	45	0.77	0.44	0.18 (0.08, 0.28)
Cakes, muffins	96	64	41	0.87	0.53	0.33 (0.24, 0.43)
Ice-cream	94	60	53	0.78	0.61	0.40 (0.31, 0.50)
Salted nuts	94	32	3	1.00	0.70	0.43 (0.33, 0.52)
TOTAL SNACKS		70	51	0.90	0.44	0.41 (0.34, 0.48)
Fizzy drinks (sodas)	95	66	58	0.84	0.58	0.45 (0.36, 0.54)
Fruit drinks	96	58	34	0.67	0.46	0.06 (-0.04, 0.16)
Diet fizzy drinks	94	45	27	0.80	0.68	0.43 (0.33, 0.52)
Sports drinks	94	24	14	0.77	0.84	0.66 (0.58, 0.74)
TOTAL DRINKS		48	33	0.77	0.64	0.40 (0.30, 0.49)

PABAK statistics were relatively similar across the four food/drink categories and indicated fair to moderate performance of the HFAI-C at the category level, with PABAKs ranging from 0.31 (95 % CI: 0.25, 0.37) for vegetables to 0.44 (95 % CI: 0.40, 0.49) for fruits. PABAK values were lowest for greens (PABAK -0.09, 95 % CI: -0.19, 0.02) and highest for grapefruit (PABAK 0.77, 95 % CI: 0.71, 0.83).

Table 3 shows the distribution of mean participant and researcher responses as well as the weighted kappa statistics for the four food/drink categories using the two, three, and four quantities of availability. Prevalence as reported by participants tended to be higher than that reported by researchers, except for the large category and for medium drinks. Kappa statistics for the dichotomized responses were generally greater than those found when items were recorded in four quantities of availability (except for vegetables). Additionally, the use of three quantities of availability for the fruit and vegetable categories was more valid than the use of dichotomized responses. The validity of the HFAI-C was fair to moderate across the two, three, and four quantities of availability. However, the validity of the drink category was only fair when the three and four quantities of availability were used, and the validity of the snack category was only fair when the four quantities of availability were used.

**Table 3:** Criterion validity comparing researcher administered and participant reported data for analytic sample using 2, 3, and 4 response categorizations^a^ (*n* = 97)

	Prevalence by participant (%)				Prevalence by researcher (%)				Weighted kappa^b^		
Category	Absent	Small	Medium	Large	Absent	Small	Medium	Large	2	3	4
Fruits	50	15	24	12	73	8	12	6	0.44 (0.40, 0.48)	0.45 (0.41, 0.50)	0.40 (0.36, 0.44)
Vegetables	36	23	24	17	61	8	11	20	0.35 (0.30, 0.40)	0.48 (0.42, 0.53)	0.41 (0.36, 0.45)
Snacks	30	35	23	12	49	4	8	39	0.41 (0.34, 0.47)	0.35 (0.30, 0.41)	0.26 (0.22, 0.31)
Drinks	52	39	7	3	67	12	13	8	0.39 (0.30, 0.48)	0.26 (0.18, 0.33)	0.25 (0.18, 0.32)

^a^Categories are 2 (absent, present), 3 (absent, small, medium/large), and 4 (absent, small, medium, large)

^b^Cicchetti-Allison linear-weighted kappa

The potential impact of ethnicity was examined by comparing validity data between homes of White British and Pakistani origin. Results were similar between groups with a few exceptions: Agreement was higher for fruits in the White British (PABAK 0.58, 95 % CI 0.53, 0.64) compared to the Pakistani (PABAK 0.32, 95 % CI 0.24, 0.39). Conversely, homes with participants of Pakistani origin had higher validity in the reporting of the quantity of vegetables (PABAK 0.34, 95 % CI: 0.28, 0.42) compared to home with White British participants (PABAK 0.31, 95 % CI: 0.22, 0.39) (See Additional file 1).

### Test-retest reliability

Results from the reliability analyses are presented in Table 4. ICCs are presented only for the dichotomized responses. When all participants with HFAI-C data available at both time points (with repeated completion within 5 months) were included in the analysis, ICCs at the category level indicate that agreement between the two time points was slight to fair, with ICCs ranging from 0.38 (95 % CI 0.29, 0.48) for drinks to 0.52 (95 % CI 0.47, 0.56) for fruits. For individual items, ICCs ranged from 0.14 (for ‘other fresh vegetables’) to 0.61 (for grapes and canned tomatoes). Restricting the sample to those who completed both assessments within a 30-day time period improved the reliability such that agreement at the category level was fair to moderate, with ICCs ranging from 0.48 (95 % CI 0.34, 0.60) for drinks to 0.58 (95 % CI 0.51, 0.64) for fruits. The ICCs for all sets of twins in BiB1000 (where both questionnaires were completed on the same day) had the smallest range of ICCs at the item level, with only four items, (tinned fruits (water/juice and syrup), grapes, and celery resulting) in ICCs less than 1.00. Agreement was substantial for all categories among the sample of twins.

**Table 4:** Test re-test reliability for full (*n* = 95), 30-day restricted (*n* = 43), and twin (*n* = 15) samples using dichotomized responses (presence/absence)

	Intra-class correlation coefficient					
Item	*n*	Full	*n*	30 days	*n* ^a^	Twins
Apples	95	0.36	43	0.31	15	1.00
Bananas	94	0.59	42	0.79	15	1.00
Grapes	92	0.61	40	0.59	15	0.65
Oranges	93	0.44	41	0.49	15	1.00
Dried fruit	93	0.47	42	0.65	15	1.00
Berries	91	0.18	40	0.28	15	1.00
Pears	93	0.46	42	0.56	15	1.00
Canned fruit, water/juice	93	0.40	42	0.37	15	0.85
Melon	89	0.38	41	0.42	15	1.00
Canned fruit, syrup	92	0.18	42	0.25	15	0.85
Plums	93	0.28	42	0.33	15	1.00
Kiwis	93	0.30	42	0.46	15	1.00
Peaches	91	0.23	42	0.42	15	1.00
Pineapple	94	0.50	43	0.54	15	1.00
Fruit salad	94	0.22	43	0.28	15	1.00
Grapefruit	94	0.16	43	0.16	15	1.00
TOTAL FRUIT		0.52 (0.47, 0.56)		0.58 (0.51, 0.64)		0.97 (0.94, 1.00)
Carrots	94	0.45	43	0.76	15	1.00
Peas	92	0.37	41	0.36	15	1.00
Tomatoes, fresh	95	0.47	43	0.55	15	1.00
Lettuce	93	0.44	42	0.41	15	1.00
Greens	93	0.35	43	0.50	15	1.00
Tomatoes, can	91	0.61	42	0.55	15	1.00
Sweet corn	93	0.33	42	0.59	15	1.00
Broccoli	91	0.54	42	0.47	15	1.00
Other vegetables, fresh	82	0.14	41	0.27	15	1.00
Cabbage	93	0.32	43	0.22	15	1.00
Green beans	95	0.39	43	0.43	15	1.00
Celery	92	0.41	42	0.47	15	0.83
TOTAL VEGETABLES		0.49 (0.44, 0.55)		0.52 (0.45, 0.60)		0.99 (0.96, 1.00)
Biscuits (cookies)	94	0.30	42	0.82	15	1.00
Crisps (chips)	94	0.36	43	0.54	15	1.00
Chocolate	92	0.21	42	0.23	15	1.00
Sweets (candies)	93	0.29	42	0.27	15	1.00
Cakes, muffins	94	0.33	43	0.44	15	1.00
Ice-cream	92	0.31	43	0.47	15	1.00
Salted nuts	92	0.29	42	0.43	15	1.00
TOTAL SNACKS		0.42 (0.35, 0.49)		0.52 (0.41, 0.61)		1.00 (1.00, 1.00)
Fizzy drinks (sodas)	93	0.37	43	0.50	15	1.00
Fruit drinks	94	0.16	43	0.11	15	1.00
Diet fizzy drinks	92	0.51	41	0.71	15	1.00
Sports drinks	93	0.36	42	0.56	15	1.00
TOTAL DRINKS		0.38 (0.29, 0.48)		0.48 (0.34, 0.60)		1.00 (1.00, 1.00)

^a^ *n* refers to # of sets of twins

## Discussion

On the whole, the validity and reliability of the 39-item HFAI-C was moderate, with high levels of sensitivity and reliability and fair to moderate agreement between participant- and researcher- collected data. The instrument was more accurate in the assessment of the presence of foods in the home compared to determining the amount available, although this had little impact on the overall validity results here. Our evaluation also indicated that the HFAI-C may be appropriate for use in households of both Whites and South Asians living in Britain.

The level of agreement we found in item-level validation is consistent with previous literature in which researcher conducted exhaustive inventories were used as a gold standard comparator [6–9, 33]; however, comparison of mean category level validation values with other studies is difficult, as others have not conducted a bootstrapping method to enable category level means with confidence intervals. Furthermore, not all previous studies have adjusted analyses and determined PABAK statistics. Boles and colleagues validated a 126-item checklist of foods and drinks in 25 homes and reported kappa values ranging from 0.03 to 1.00 for fruits and vegetables [33]. Mean category level kappas were not provided. Similarly, a validation study by Marsh et al. [9] presented item level Cohen’s kappa statistics for 31-items within the categories of fruit juices, fruits and vegetables. Ranges within categories were similar to PABAK results of the current study; with kappa values for fruit juices ranging from 0.24–0.53; kappa values for fruits at 0.12–0.76 (compared to our PABAK values ranging from 0.17–0.77); and kappa values for vegetables between 0.19–0.66 (compared to our PABAK values ranging from 0.01–0.66). These ranges fit into similar boundaries of acceptability (e.g. the majority of fruit items from both studies were described as having at least moderate validity (0.41–0.60). However, caution should be applied when comparing adjusted PABAK findings with kappa statistics as adjusted analyses using PABAK resulted in higher levels of agreement in all studies, including the present study.

In a further validation study of the Healthy Home Survey by Bryant et al. [10] mean PABAK values for categories were provided, although confidence intervals were not. This study differed in the type of data collected as, participants reported all items within 6 food and drink categories (i.e. using an exhaustive ‘within category’ approach rather than a checklist). Mean PABAK values were higher than the current study (0.85 and 0.85 for fresh fruits and vegetables respectively) although these were simple means and did not include category level variables. Confidence intervals were generated for the current study to provide a description of the distribution of PABAK data. These results indicated a substantial variation between item validation results and mean confidence interval values ranged from -0.02 to 0.87. This suggests that the ability of the checklist to accurately reflect what was found in homes by researchers varied between households. Other validation studies including those by Crockett et al. [6], Miller and Edwards [7] and Fulkerson et al. [8] report similar sensitivity and specificity results to those of the HFAI; however Kappa values were higher. This might be explained by the differences in study design as, instead of comparing checklist data to exhaustive researcher conducted inventories, these other studies validated their participant completed checklists by comparing data to repeated administration of the checklists by researchers.

Test-re-test data suggest that the HFAI-C demonstrated fair reliability, particularly if the two repeated administrations were within 30 days of each other. Given the high probability that there were actual changes to foods in the home over this period, the relatively lower reliability with up to 5 months between measurements may actually indicate the transient nature of the environment and acts as a measure of stability rather than consistency. Conversely, when administrations occurred in the same day (i.e. by parents with twins), the higher reliability estimates suggested that the tool has substantial consistency.

It is a limitation that our validity data may have been impacted by differences in the time referent of the HFAI-C compared to the researcher-conducted inventory. Whereas the HFAI-C required participants to report the maximum amount of each item over the past 7 days, researchers conducted inventories on a single day. Given this difference, it is not surprising that, for the most part, prevalence as reported by participants was higher than that reported by researchers. This issue has been reported previously by Marsh et al., whose checklist required participants to report over the previous 7 days, whereas researcher conducted inventories were on a single day [9]. These authors theorised that the variability in observed agreement statistics may therefore be related to the perishable nature of some foods. However, kappa and PABAK statistics in the current study were similar between perishable and non-perishable items. Evidence from a small study with 9 households, each measured five times, indicates high intra-monthly changes in availability due to purchasing and consumptions behaviours [34]. However, in larger studies home food availability has been shown to be relatively stable, as the within household variation of home food availability is relatively low compared to between household variability [35]. Intuitively, this is logical, as households tend to purchase similar items over time. In fact, research in this area suggests that, only one observation per household may be required to obtain a correlation between *r* = 0.7–0.9 with the true within household mean when assessing the number of items available [35]. Nevertheless, it is likely that the absolute maximum amount of foods reported amount over 7 days will differ from that in a single day within a household, especially with fresh produce. This may be particularly important in low-income households who are less likely to have car access enabling them to shop at larger retailers [36].

It is a strength of the current study that we assessed the quantity of foods and drinks that were available. We found that quantitative results were generally less valid than the identification of the presence or absence of items. However, these differences had little impact on the overall validity results. Measurement of the presence/absence of food items in the home is the most common approach used in other checklists of home food availability, perhaps because of feasibility. Data from this study may indicate that such an approach is most appropriate for the assessment of home food availability when using a self-report checklist. It has been argued that the quantity of foods available provides a richer picture of the environment and would better discriminate between households providing a ‘healthy’ compared to a ‘non-healthy’ environment [10]. Further research is required in this area as few checklists assess any level of quantity other than presence or absence.

It is a strength that this study included participants with South Asian cultural identities. Given the large role of culture in food selection types of foods and drinks available in the home are likely to be impacted and differences were anticipated between White and South Asians in our sample. In the checklist development stage, data from 24-h dietary recalls taken from South Asian populations in Bradford were examined to identify foods or drinks that were consumed regularly that were missing from the checklist. Instead of identifying completely new items, this process identified prompts and examples that needed to be added. For example, under ‘other vegetables’, ‘okra’ was included as one of the examples. Providing a range of ethnically appropriate examples or prompts for measures is therefore one way that a tool may be developed or modified for use in mixed population groups. Other approaches to make tools more culturally have been suggested by others including working with the target populations to modify existing tools (i.e. delete items, add items, or altering categories) or by applying modifications (i.e. additions) to produce a tool appropriate for use in a mixed population [12, 37, 38]. The latter approach ensures appropriate items are included, but increases the length of checklists, which may be less feasible to administer. Administration of the HFAI-C in other populations should involve working with members of the intended population to modify the prompt and examples provided without increasing the number of items.

It is possible that the validity of such measures may be impacted by cultural or social identity (for example, participants of one ethnicity may be more accurate at reporting availability than others using the checklist). Our investigation of this identified similarities in validity and reliability by ethnicity, except that agreement between participant and researcher reported fruit availability was greater for White British participants compared to those of Pakistani origin, but that participants of Pakistani origin had better validity for reporting the quantity of vegetables in their homes. This study was not statistically powered to identify differences by ethnicity, but analyses were conducted as a means to explore potential disparities in the validity and reliability. Homes with participants of Pakistani origin from this cohort have a greater amount of fruits available compared to those of White British origin [23], which may be one possible explanation for differences in the ability to accurately measure them; as the measurement of fresh produce is more likely to be impacted by shopping and consumption behaviours [36] Further research may be warranted to explore potential cultural or ethnic difference in reporting accuracy, in addition to understanding the impact of statistical adjustment of other potential covariates [35].

It has been argued that the use of checklists compared to researcher-conducted exhaustive inventories is somewhat analogous to differences between assessment of diet using food frequency questionnaires (FFQs) and food diaries or histories [35]. The former are restricted to the items that are to be relevant to the investigators’ hypotheses, with the latter able to collect an exhaustive or complete account. FFQs are useful in large epidemiological studies in which detailed dietary measurement is not feasible or required, and the data ranks individuals moderately well along a distribution of intake, but absolute amounts are not well-quantitated [39]. Similarly, a home food availability checklist is a less time intensive method that is likely to be more feasible in large epidemiological studies, and it can be useful if ranking individuals on pre-determined types of foods is adequate to answer the study questions. Research has attempted to quantify the absolute amount of nutrients available in the home using barcode scanners [15, 22]. This method requires a researcher or the participant to scan all foods and drinks in their homes and is labour intensive. More recently, the ability to quantify nutrient availability in the home has been tested by comparing detailed home food inventory data with data gathered from existing Food Frequency Questionnaires (Block Dietary Fat Screener and Block Fruit–Vegetable–Fiber Screener) [40]; with observed high correlations between the two (e.g. *r* = 0.76–0.78, *p* < 0.01 for the Fruit-Vegetable-Fiber Screener). Annotated grocery receipts may also be used to quantify home food availability [41] although this method relies on detailed receipts (or participant reporting on ‘vague’ receipts, ignores foods such as those gathered from gardens and its analysis and interpretation can be labour intensive). Methods that do not rely on self-reported data, including researcher conducted inventories, are most likely to provide the most valid assessment; however these are often less feasible especially in larger studies.

This study adds to the literature on the measurement of home food availability by providing an evaluation of a brief checklist in relatively large a bi-ethnic sample, that can be used to rank the availability of foods and drinks (including an estimation of quantity) that are hypothesised to relate to childhood obesity. An important strength of the current study is its inclusion of a diverse ethnic population with high levels of deprivation. The HFAI-C has also been administered to a wider sample of 1700 families within the BiB1000 cohort, which benefits from the inclusion of a large amount of other assessments [42]. This will allow further examination of home food availability in relation to exposures such as ethnicity and outcomes including behavioural, social and health outcomes; thus providing further insight into how the home food environment may relate to obesity and health related behaviours. One limitation of the validity of the tool might relate to confusion in the interpretation on some of the items. Items with the lowest validation results were ‘greens’ and ‘other fresh vegetables’. It is likely that lower values for these items related to uncertainties in their definitions as we have since learnt that this differs between individuals. Cognitive testing of the HFAI-C was not conducted due to its checklist nature, although issues related to interpretation may have been alleviated if cognitive testing was conducted. Further implementation of the HFAI-C should therefore provide a clearer definition of these items. Like other checklists, the HFAI-C is limited by the foods that are include in the list and therefore cannot assess the overall availability of foods in the home. Lastly, although the variance in the timing between administrations for the reliability study permitted a greater understanding of the impact of time on measurement, estimates may have been improved if data were gathered from a larger sample of participants in which data were collected within 30 days or even 7 weeks of one another.

## Conclusion

The HFAI-C is a simple checklist that can be used to assess the availability of foods in the home, in addition to estimating the amounts available. Similar to other home food availability checklists, the HFAI-C demonstrated fair to moderate validity and reliability, with high variability between items. More research is recommended in this area to confirm this hypothesis or to improve on the validity of the measurement of quantity. Future work should also focus on the ability of the checklist to discriminate between participant characteristics such as obesity and to evaluate the impact of home food availability on behaviours such as dietary intake.

## Additional file

Additional file 1: Table S1. White British (WB, *n* = 47) and Pakistani (P, *n* = 40) criterion validity for analytic sample using 2, 3, and 4 response categorizations* (*n* = 97). Table S2. White British (WB) and Pakistani (P) reliability for full (*n* = 95), 30-day restricted (*n* = 43), and twin (*n* = 15) samples using dichotomized responses (presence/absence). Table S3. Comparison of weighted kappa statistics for 2, 3, and 4 response categorizations using simple averaging vs. category-level variables. Table S4. Comparison of ICC for simple averages vs. category-level variables. (DOCX 18 kb)
