# Supplementary material for: Validity and reliability of the semi-quantitative self-report Home Food Availability Inventory Checklist (HFAI-C) in White and South Asian populations
Source: Int J Behav Nutr Phys Act. 2016 May 4;13:56. doi: 10.1186/s12966-016-0381-y (PMC4857434; doi:10.1186/s12966-016-0381-y)
Supplement: Additional file 1: Table S1. — White British (WB, n = 47) and Pakistani (P, n = 40) criterion validity for analytic sample using 2, 3, and 4 response categorizations* (n = 97). Table S2. White British (WB) and Pakistani (P) reliability for full (n = 95), 30-day restricted (n = 43), and twin (n = 15) samples using dichotomized responses (presence/absence). Table S3. Comparison of weighted kappa statistics for 2, 3, and 4 response categorizations using simple averaging vs. category-level variables. Table S4. Comparison of ICC for simple averages vs. category-level variables. (DOCX 18 kb) [file 12966_2016_381_MOESM1_ESM.docx]

**Additional file 1**

TABLE S1. White British (WB, *n*=47) and Pakistani (P, *n*=40) criterion validity for analytic sample using 2, 3, and 4 response categorizations* (*n*=97)

|  | **PABAK** | |  | **Weighted Kappa**† | | | | | | | |
| --- | --- | --- | --- | --- | --- | --- | --- | --- | --- | --- | --- |
|  |  |  |  | **2 categories** | |  | **3 categories** | |  | **4 categories** | |
| **Category** | **WB** | **P** |  | **WB** | **P** |  | **WB** | **P** |  | **WB** | **P** |
| Fruits | 0.58 (0.53, 0.64) | 0.32 (0.24, 0.39) |  | 0.53 (0.47, 0.59) | 0.37 (0.31, 0.43) |  | 0.49 (0.41, 0.55) | 0.42 (0.36, 0.48) |  | 0.45 (0.39, 0.52) | 0.36 (0.30, 0.41) |
| Vegetables | 0.31 (0.22, 0.39) | 0.34 (0.26, 0.42) |  | 0.34 (0.26, 0.41) | 0.39 (0.33, 0.45) |  | 0.42 (0.34, 0.49) | 0.56 (0.48, 0.64) |  | 0.34 (0.28, 0.40) | 0.48 (0.41, 0.55) |
| Snacks | 0.49 (0.39, 0.57) | 0.34 (0.23, 0.46) |  | 0.47 (0.38, 0.56) | 0.35 (0.26, 0.45) |  | 0.33 (0.27, 0.41) | 0.37 (0.28, 0.47) |  | 0.23 (0.19, 0.29) | 0.28 (0.21, 0.36) |
| Drinks | 0.41 (0.28, 0.54) | 0.41 (0.27, 0.56) |  | 0.35 (0.20, 0.49) | 0.43 (0.29, 0.57) |  | 0.29 (0.16, 0.42) | 0.20 (0.09, 0.33) |  | 0.29 (0.17, 0.41) | 0.21 (0.11, 0.32) |

*Categories are 2 (absent, present), 3 (absent, small, medium/large), and 4 (absent, small, medium, large)

†Cicchetti-Allison linear-weighted kappa

TABLE S2. White British (WB) and Pakistani (P) reliability for full (*n*=95), 30-day restricted (*n*=43), and twin (*n*=15) samples using dichotomized responses (presence/absence)

|  |  | **ICC** | | | | | | | |
| --- | --- | --- | --- | --- | --- | --- | --- | --- | --- |
|  |  | **Full sample** | |  | **30 days** | |  | **Twins** | |
| **Category** |  | **WB** (*n*=45) | **P** (*n*=40) |  | **WB** (*n*=18) | **P** (*n*=20) |  | **WB** (*n*=6) | **P** (*n*=9) |
| Fruits |  | 0.55 (0.49, 0.61) | 0.47 (0.39, 0.54) |  | 0.66 (0.58, 0.75) | 0.49 (0.39, 0.59) |  | 0.94 (0.85, 1.00) | 1.00 (1.00, 1.00) |
| Vegetables |  | 0.46 (0.38, 0.54) | 0.54 (0.46, 0.61) |  | 0.52 (0.40, 0.65) | 0.57 (0.46, 0.67) |  | 1.00 (1.00, 1.00) | 0.98 (0.92, 1.00) |
| Snacks |  | 0.44 (0.34, 0.55) | 0.38 (0.26, 0.50) |  | 0.60 (0.43, 0.75) | 0.45 (0.28, 0.60) |  | 1.00 (1.00, 1.00) | 1.00 (1.00, 1.00) |
| Drinks |  | 0.36 (0.22, 0.49) | 0.35 (0.20, 0.50) |  | 0.52 (0.31, 0.72) | 0.38 (0.17, 0.58) |  | 1.00 (1.00, 1.00) | 1.00 (1.00, 1.00) |

TABLE S3. Comparison of weighted kappa statistics for 2, 3, and 4 response categorizations using simple averaging vs. category-level variables

| **Category** |  | **2 categories** | | |  | **3 categories** | | |  | **4 categories** | | |
| --- | --- | --- | --- | --- | --- | --- | --- | --- | --- | --- | --- | --- |
|  |  | Simple average of results | Category variable | Category variable – Bootstrapped CI |  | Simple average of results | Category variable | Category variable – Bootstrapped CI |  | Simple average of results | Category variable | Category variable – Bootstrapped CI |
| Fruits |  | 0.29 | 0.44  (0.40, 0.48) | 0.44  (0.40, 0.48) |  | 0.26 | 0.45  (0.41, 0.50) | 0.45  (0.41, 0.50) |  | 0.25 | 0.40  (0.36, 0.43) | 0.40  (0.36, 0.44) |
| Vegetables |  | 0.31 | 0.35  (0.30, 0.40) | 0.35  (0.30, 0.40) |  | 0.27 | 0.48  (0.43, 0.53) | 0.48  (0.42, 0.53) |  | 0.26 | 0.41  (0.36, 0.45) | 0.41  (0.36, 0.45) |
| Snacks |  | 0.26 | 0.41  (0.34, 0.47) | 0.41  (0.34, 0.47) |  | 0.18 | 0.35  (0.30, 0.41) | 0.35  (0.30, 0.41) |  | 0.15 | 0.26  (0.21, 0.31) | 0.26  (0.22, 0.31) |
| Drinks |  | 0.35 | 0.39  (0.30, 0.48) | 0.39  (0.30, 0.48) |  | 0.22 | 0.26  (0.17, 0.34) | 0.26  (0.18, 0.33) |  | 0.22 | 0.25  (0.18, 0.32) | 0.25  (0.18, 0.32) |

TABLE S4. Comparison of ICC for simple averages vs. category-level variables

|  |  | **ICC** | | | | | | | |
| --- | --- | --- | --- | --- | --- | --- | --- | --- | --- |
|  |  | **Full sample** | |  | **30 days** | |  | **Twins** | |
| **Category** |  | Simple average | Category variable – Bootstrapped CI |  | Simple average | Category variable – Bootstrapped CI |  | Simple average | Category variable – Bootstrapped CI |
| Fruits |  | 0.36 | 0.52 (0.47, 0.46) |  | 0.43 | 0.58 (0.51, 0.64) |  | 0.96 | 0.97 (0.94, 1.00) |
| Vegetables |  | 0.40 | 0.49 (0.44, 0.55) |  | 0.46 | 0.52 (0.45, 0.60) |  | 0.99 | 0.99 (0.96, 1.00) |
| Snacks |  | 0.30 | 0.42 (0.35, 0.49) |  | 0.46 | 0.52 (0.41, 0.61) |  | 1.00 | 1.00 (1.00, 1.00) |
| Drinks |  | 0.35 | 0.38 (0.29, 0.48) |  | 0.47 | 0.48 (0.34, 0.60) |  | 1.00 | 1.00 (1.00, 1.00) |
